# Development of predictive nomograms clinical use to quantify the risk of diabetic foot in patients with type 2 diabetes mellitus

**DOI:** 10.3389/fendo.2023.1186992

**Published:** 2023-06-14

**Authors:** Bocheng Peng, Rui Min

**Affiliations:** ^1^ Department of Pain, Wuhan Fourth Hospital, Wuhan, China; ^2^ Department of Geriatrics, Wuhan Fourth Hospital, Wuhan, China

**Keywords:** diabetic foot, nomogram model, prediction, risk factors, type 2 diabetes mellitus

## Abstract

**Objective:**

The aim of the study was to explore the risk factors for diabetic foot disease in patients with type 2 diabetes mellitus and to establish and verify the nomogram model of DF risk in patients with T2DM.

**Methods:**

The clinical data of 705 patients with type 2 diabetes who were hospitalized in our hospital from January 2015 to December 2022 were analyzed retrospectively. According to random sampling, the patients were divided into two groups: the training set (DF = 84; simple T2DM = 410) and the verification set (DF = 41; simple T2DM = 170). Univariate and multivariate logistic regression analysis was used to screen the independent risk factors for DF in patients with T2DM in the training set. According to the independent risk factors, the nomogram risk prediction model is established and verified.

**Results:**

Logistic regression analysis showed age (OR = 1.093, 95% CI 1.062–1.124, P <0.001), smoking history (OR = 3.309, 95% CI 1.849–5.924, P <0.001), glycosylated hemoglobin (OR = 1.328, 95% CI 1.173–1.502, P <0.001), leukocyte (OR = 1.203, 95% CI 1.076–1.345, and LDL-C (OR = 2.002, 95% CI 1.463–2.740), P <0.001) was independent risk factors for T2DM complicated with DF. The area of the nomogram model based on the above indexes under the ROC curve of the training set and the verification set is 0.827 and 0.808, respectively; the correction curve shows that the model has good accuracy; and the DCA results show that when the risk threshold is between 0.10–0.85 (training set) and 0.10–0.75 (verification set), the clinical practical value of the model is higher.

**Conclusion:**

The nomogram model constructed in this study is of high value in predicting the risk of DF in patients with T2DM and is of reference value for clinicians to identify people at high risk of DF and provide them with early diagnosis and individual prevention.

## Introduction

Over the past decades, the rapidly increasing prevalence and incidence of diabetes mellitus have become major public health issues worldwide. In 2015, it was estimated that around 415 million people are living with diabetes worldwide, according to the International Diabetes Federation, and this number continues to grow and has been projected to grow up to 642 million by 2040 at the same time, with a growth rate of 55% over the next 20 years (1). Epidemiological studies have shown that the incidence of diabetic foot (DF) (2) in diabetic patients ranges from 15% to 25% in their lifetime, and it is the leading cause of non-traumatic amputation (3). DF is a serious complication of diabetes, which is closely related to peripheral vascular, neuropathy, and increased mechanical pressure of the foot (4). If the patients with DF are not treated in time, the disease will progress, resulting in lower limb ulcers, gangrene, and osteomyelitis, which will lead to more serious adverse outcomes such as amputation or even death. The quality of life of type 2 diabetic patients with diabetic foot was significantly decreased, and their socio-economic burden was significantly increased (5). However, the early clinical manifestations of DF patients are not obvious, and there are some difficulties in diagnosis. In view of the possible serious harm of diabetic foot, we urgently need to put forward an effective evaluation method to predict the occurrence of DF in the early stages (6). Previous studies (7) have mainly focused on the risk factors for DF, but there is no intuitive and simple method to more accurately assess the risk of diabetic foot in patients with type 2 diabetes. As a forecasting tool, the nomogram model can show the corresponding relationship between a specific disease and risk factors through an intuitive graphical scoring system and accurately predict the risk of disease occurrence (8). Based on the above advantages, the purpose of this study is to build a nomogram model to predict the risk of DF in patients with T2DM, to guide clinicians in identifying people at high risk of DF, and to provide them with early diagnosis and individualized prevention.

## Materials and methods

### Study design and participants

To solve this clinical problem, we followed the methods of the article we published earlier (9) and improved them. We designed and implemented a retrospective study, and 705 patients with T2DM who were hospitalized in Wuhan Fourth Hospital and Zhongnan Hospital from January 2015 to December 2022 were included in this study at all. Some of the patient data came from the data collected in our previous study (9), but most of the population data for patients with diabetic foot and type 2 diabetes mellitus were derived from the collected data. There were 461 males with an average age of 55.70 ± 11.83 years and 244 females with an average age of 64.52 ± 11.39 years. With reference to the method of Jiang et al. (10), according to the principle of random sampling, the cases were divided into two groups: the training set (n = 494, including 84 T2DM patients with DF and 410 simple T2DM patients) and the verification set (n = 211, including 41 T2DM patients with DF and 170 simple T2DM patients). The ratio between them was 7:3. Criteria for selecting the participants were as follows: Inclusion criteria: (1) All patients met the diagnostic criteria for type 2 diabetes published by the 1999 WHO diagnostic criteria; (2) the age of patients was over 18 years; and (3) all patients gave informed consent to this study. Exclusion criteria: (1) Type 1 DM patients or secondary DM patients; (2) patients with severe hepatic and renal dysfunction; (3) diabetic patients during pregnancy and lactation; (4) patients with malignant tumors or acquired immune dysfunction; and (5) patients with a severe lack of case data. This study is in line with the Helsinki Declaration and has been approved by the Ethics Committee of Wuhan Fourth Hospital (KY2023-032-01).

### Data collection

A clinical investigation case report form (CRF) we designed was used to collect the clinical data of the patients from the Hospital Information System (HIS) system of the hospital. We collected demographic data of the patients including, gender, age, BMI, diabetes course, smoking history, etc.; results of the first biochemical examination of fasting venous blood after admission, including fasting blood glucose (FBG), glycated hemoglobin (HbA1c), white blood cell (WBC), albumin (ALB), triglyceride (TG), blood uric acid (BUA), high density lipoprotein cholesterol (HDL-C), low density lipoprotein cholesterol (LDL-C), and fibrinogen (FIB). Results of an ultrasonographic examination of atherosclerosis or plaque in the lower extremities of patients. We set up an epidata database to collect the required survey data according to the CRF, and following data input, a parallel check of all data by two people was performed to control the quality of the data.

### Statistical analysis

Continuous variables with a normal distribution and non-normal distribution were expressed as “mean ± standard deviation (x ± s)” and “the median (lower quartile, upper quartile) [M (P25p75)]”, respectively. The T-test was used for continuous variables following a normal distribution; otherwise, the Wilcoxon rank-sum test was used for continuous variables without a normal distribution. Categorical variables were expressed as percentage component ratios, and the Chi-square test was used for comparison between groups. Two-sided tests were used for all statistical data, and p-values <0.05 were considered statistically significant. Confidence intervals (CI) were all set at 95%. All statistical analysis was carried out using SPSS 26.0 software.

### Establishment and verification of nomogram model

Firstly, the risk factors of T2DM complicated with DF were screened by univariate logistic regression analysis, and then the selected risk factors were introduced into multivariate logistic regression analysis to determine the independent risk factors of DF in patients with T2DM. R4.2 software was used to establish a nomogram prediction model based on all independent risk factors for DF in patients with T2DM. The accuracy of the model was tested by using a calibration curve containing 2,000 bootstrap samples, and the nomogram prediction model was validated internally and externally. The area under the curve (AUC) of the receiver operating characteristic curve was used to estimate the performance of a nomogram model. Furthermore, decision curve analysis (DCA) quantifies the net benefit to patients with T2DM at different threshold probabilities, and the clinical application value of the nomogram was determined. All the statistical analyses were performed with R4.2 and the corresponding ROCR, rms, and ggDCA packages.

## Results

### Baseline clinical characteristics of participants

A total of 705 patients with T2DM were included in this study, with an average age of 57.72 ± 12.00 years old. Among them, there were 494 patients with T2DM in the training set, with an average age of 57.17 ± 11.62 years, of whom 66.19% were male, and 211 patients with T2DM in the verification set, with an average age of 59.01 ± 12.76 years, of whom males account for 63.51%. In the statistical analysis, there was a significant difference in serum BUA level between T2DM patients in the training set and verification set (p <0.05); there was no significant difference in other clinical indexes between the two groups (p >0.05), as shown in **Table 1**.

**Table 1:** Differences in characteristics between the training set group and the verification set group.

Variables	Verification set (n = 211)	Training set (n = 494)	X^2^/Z/T-value	P-value
Gender (male/female)	134/77 (63.51%)	327/167 (66.19%)	0.472	0.492
Age (years)	59.01 ± 12.76	57.17 ± 11.62	1.872	0.062
BMI (kg/m^2^)	24.96 (22.46, 27.10)	24.78 (22.49, 27.06)	−0.156	0.876
Course of T2DM (years)	8.00 (3.00, 10.00)	7.00 (3.00, 10.00)	−1.172	0.241
History of smoking	65/146 (30.81%)	174/320 (35.22%)	1.287	0.257
atherosclerosis	85/125 (40.28%)	203/291 (41.09%)	0.023	0.879
HbA1c (%)	8.60 (7.10, 10.60)	8.25 (6.90, 10.00)	−1.673	0.094
FBG (mmol/L)	9.18 (6.94, 12.63)	9.30 (7.01, 12.30)	−0.093	0.926
WBC (10^9^/L)	5.86 (4.59, 7.33)	6.35 (4.72, 7.97)	−1.468	0.142
ALB (g/L)	34.88 ± 5.48	35.79 ± 5.29	−2.080	0.038
BUA (mmol/L)	333.60 (256.80, 406.70)	332.40 (276.38, 398.95)	−0.641	0.521
TG (mmol/L)	1.44 (1.03, 2.46)	1.14 (0.88, 1.37)	−7.882	<0.001
HDL-C(mmol/L)	0.98 (0.84, 1.17)	0.98 (0.83, 1.17)	−0.247	0.784
LDL-C (mmol/L)	2.65 (2.06, 3.20)	2.65 (2.03, 3.24)	−0.148	0.883
FIB (mg/dl)	400.28 (354.56, 443.47)	394.37 (358.99, 443.19)	−0.029	0.977

### Univariate and multivariate logistic regression analysis

The variables with statistically significant differences in training sets were introduced into the univariate logistic regression analysis. The results showed that age, course of diabetes, history of smoking, lower extremity atherosclerosis, or plaque, HbA1c, WBC, and LDL-C were the risk factors of DF in patients with T2DM (p <0.05), while BUA was the protective factor of DF in patients withT2DM (p <0.05). LDL-C were the risk factors for DF in T2DM patients (p <0.05), while BUA was a protective factor for DF in T2DM patients (p <0.05). With DF in T2DM patients as the dependent variable, the above six risk factors (age, course of diabetes, history of smoking, lower extremity atherosclerosis or plaque, HbA1C, WBC, and LDL-C) were put into multiple factors logistic regression analysis. The inclusion and exclusion criteria for independent variables were 0.05. Forward stepwise regression was used to select the variables. The results showed that only five factors, including age (OR = 1.093, 95% CI 1.062–1.124, P <0.001), history of smoking (OR = 3.309, 95% CI 1.849–5.924, P <0.001), HbA1c (OR = 1.328, 95% CI 1.173–1.502, P <0.001), WBC (OR = 1.203, 95% CI 1.076–1.345, P = 0.001), and LDL-C (OR = 2.002, 95% CI 1.463–2.740, P <0.001), became independent risk factors for DF in patients with T2DM, as shown in **Table 2**.

**Table 2:** Independent risk factors of DF in patients with T2DM.

Variables	β	SE	Wald	P	OR (95% CI)
Age	0.089	0.014	38.198	<0.001	1.093 (1.062, 1.124)
History of smoking	1.197	0.297	16.224	<0.001	3.309 (1.849, 5.924)
HbA1c	0.283	0.063	20.262	<0.001	1.328 (1.173, 1.502)
WBC	0.185	0.057	10.593	0.001	1.203 (1.076, 1.345)
LDL-C	0.694	0.16	18.806	<0.001	2.002 (1.463, 2.740)

### Establishment of a nomogram prediction model

**Figure 1:**
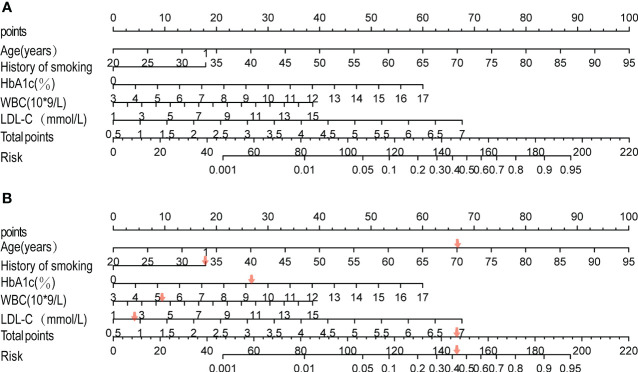
Development of the risk nomogram **(A)** and the dynamic nomogram for an example **(B)**. The nomogram to predict the risk of diabetic foot in patients with T2DM was developed with predictors including age, history of smoking, HbA1C, WBC, and LDL-C. Smoking history, 1; no smoking history, 0.

Based on the results of the multi-factor logistic regression analysis, five separate influence factors of DF in T2DM patients (age, smoking history, HbA1C, WBC, and LDL-C) were introduced into R4.2 software to establish a nomogram model for predicting DF risk in T2DM patients, as shown in **Figure 1A**. According to variables such as age, smoking history, HbA1c, WBC, and LDL-C, we can get different scores on the top score line (0–100 points); the total score is obtained by adding all the variable scores, and then the corresponding probability of DF in patients with T2DM can be found on the risk line; the higher the total score, patients with T2DM have a higher risk of developing DF. Here is an example to better understand the nomogram model: if the patient with T2DM is 70 years old and complicated by a smoking history, an HbA1c of 9.2%, a WBC of 5.21 * 10^9^/L, and an LDL-C of 2.72 mmol/L, the probability of DF is estimated to be 42% (**Figure 1B**).

### Verification of nomogram prediction model

**Figure 2:**
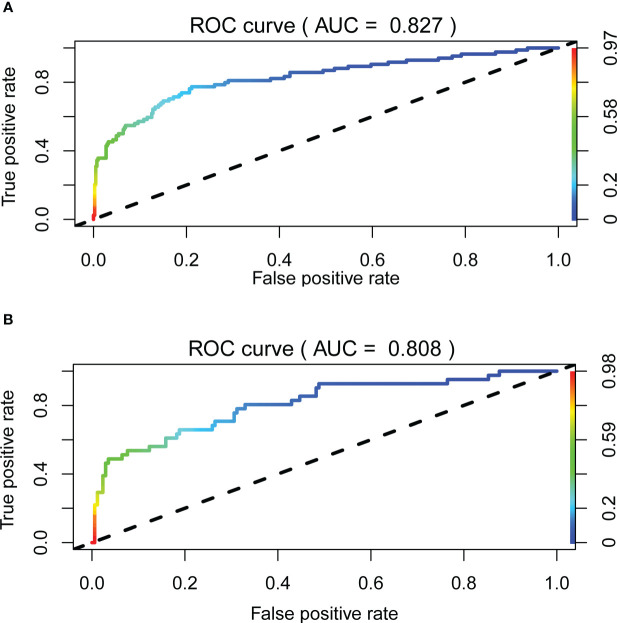
The accuracy of the nomogram for predicting DF using the ROC curve, the area under the ROC curve AUC represents the ability of the model to distinguish between DF and simple type 2 diabetes. When AUC = 0.5 said that the probability of the model distinguishing diabetic foot patients was 50%, it indicated that the probability of the model distinguishing diabetic foot patients was 100%. The closer the AUC was to 1, the stronger the distinguishing ability of the model was. The area under the ROC curve of the training set and verification set of the model (AUC) was 0.827 **(A)** and 0.808 **(B)**, respectively. It showed that the model has good distinguishing ability.

**Figure 3:**
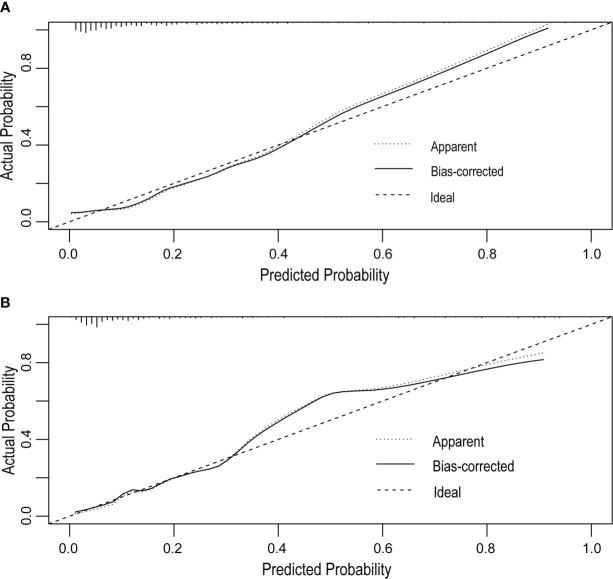
Calibration curves of the DF risk in nomogram prediction. The thick dotted line represents an ideal prediction, and the thin dotted line represents the predictive ability of the nomogram. The closer the thick dotted line fit is to the thin dotted line, the better the predictive accuracy of the nomogram is. The calibration curves of the training set **(A)** and the verification set **(B)** have good agreement between the prediction probability and the actual probability, and the average absolute errors are all less than 0.05.

**Figure 4:**
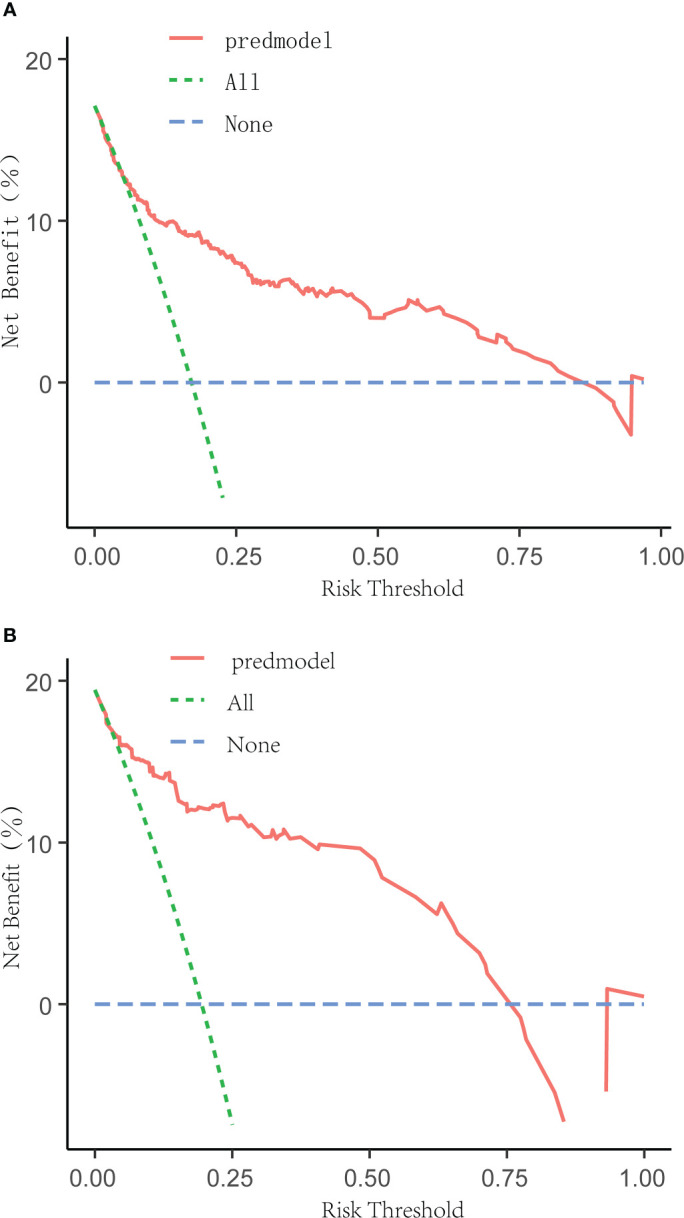
Decision curve analysis for the DF risk nomogram. The y-axis measured the net benefit. The green dotted line represented the assumption that all patients had DF. The blue dotted line represented the assumption that all patients had no DF. The solid red line represented the risk nomogram. The threshold ranges of the nomogram model in the training set **(A)** and verification set **(B)** are 0.10 to 0.85 and 0.10 to 0.75, respectively, indicating that the model has a wide range of safety and high clinical practical value.

The receiver operating characteristic (ROC) curve was used to evaluate the performance of the model in predicting the risk of DF in T2DM patients. The area under the curve (AUC) of the ROC was 0.827 in the training set (**Figure 2A**) and 0.808 in the verification set (**Figure 2B**), which indicated the model had good performance. A total of 2,000 bootstrapping samples were generated to replace the original samples, and the whole modeling process was repeated to obtain calibrated curves to verify the accuracy of the nomogram model. The calibration curve of the nomogram after correction illustrated that the model has good consistency between the predicted and observed values of the training set and the verification set, and the accuracy is high (**Figures 3A, B**). In addition, the DCA curve showed that when the risk threshold was between 0.10–0.85 (training set) and 0.10–0.75 (verification set) (**Figures 4A, B**), patients got higher clinical net benefits, indicating that the model has a wide range of safe application thresholds and high clinical practical value.

## Discussion

With the rapid growth of diabetes worldwide and the trend of aging, diabetes and its complications are becoming the focus of global public health challenges (11). In the previous literature (12), 40% to 60% of non-traumatic amputations were caused by DF, and the hospital mortality rate of DF patients can be as high as 12% (13). In clinical practice, some patients are insensitive to pain because of neuropathy, and the decrease in arterial blood flow may make local skin redness and other inflammatory phenomena not obvious, which makes it difficult to make an early diagnosis of DF and delay the time of treatment. Once the patients are complicated with infection and necrosis, the disease progresses rapidly, and clinical treatment is extremely difficult, the risk of amputation and death of the patients will be greatly increased (6). Based on the above, it is particularly important to recognize and deal with DF early and promptly. We urgently need to put forward an evaluation method that can effectively predict the occurrence of DF.

According to the statistical results in the previous research, factors such as advanced age, long course of disease, poor control of blood glucose, neuropathy and vascular disease, infection, and so on will increase the risk of DF in patients with T2DM, even the risk of amputation and death (14); however, the relevant assessment tools that can effectively forecast the risk of DF in patients with T2DM have been rarely studied previously (15, 16). Therefore, it makes sense to produce a systematic, simple, and efficient clinical tool for predicting the risk of DF in patients with T2DM. The nomogram model can synthesize multiple risk factors and predict the probability of final events graphically, and it has been widely used in various fields (17). However, there is no nomogram model to predict the risk of DF in patients with T2DM. Therefore, according to the above research, we incorporate several related potential risk factors, explore the independent risk factors for DF in patients with T2DM, and construct a nomogram model to predict the risk of DF in patients with T2DM.

Our study showed that age, smoking history, HbA1c, WBC, and LDL-C were independent risk factors for the occurrence of DF, which was consistent with the results reported in previous literature (7). Age, as an independent risk factor for the occurrence of DF, has a significantly increased risk in elderly patients, which may be related to diabetes-related complications with age, such as peripheral neuropathy and vascular disease, chronic renal insufficiency, and so on (18). Previous studies have confirmed that smoking not only increases the risk of DF (19) but also has a great risk of recurrence (20). Similar results have been obtained in this study. Smoking significantly increases the risk of DF, which may be related to the death of nerve and vascular-related cells induced by oxidative stress, the decrease of tissue blood flow and oxygen supply, and the increase in chronic inflammation (21). As we all know, long-term exposure to hyperglycemia will damage vascular endothelial cells and basement membranes, hinder the blood supply of tissue microcirculation, and thus accelerate the occurrence and development of atherosclerosis and damage to the peripheral nerves of the foot under a hypercoagulable state, resulting in local ischemia of the foot (22). In this study, HbA1c was used to reflect the control level of blood glucose in patients within 2–3 months (23), which proved that hyperglycemia plays an important role in the risk of DF in patients with T2DM. Therefore, maintaining a stable blood glucose level in patients with T2DM is of great significance to prevent the occurrence of DF (24). In addition, as a sensitive indicator of acute inflammation, the level of WBC can reflect the occurrence and outcome of inflammation. The level of WBC in patients with DF is higher than that in patients with simple T2DM and is positively correlated with the degree of infection (25, 26). Therefore, WBC can be used as one of the indexes to predict the risk of DF in patients with T2DM. We also found that the level of LDL-C is an independent risk factor for DF in patients with T2DM, which may be due to the excessive production of LDL-C and the deposition of cholesterol on the walls of peripheral arteries to form atherosclerosis (27). As a result, the blood supply to the lower extremities and feet is insufficient, and the patient is in a state of ischemia and hypoxia for a long time, resulting in tissue damage and even necrosis.

Based on the results of univariate and multivariate logistic regression analysis in the training set, we successfully constructed a nomogram model to predict the risk of DF in T2DM patients and validated the model internally and externally by using the verification set data. The correction curve was accomplished by generating 2,000 bootstrapping samples to replace the original samples and repeating the entire modeling process. The results showed that the area under the curve (AUC) of ROC in the training set was 0.827 and the area under the curve (AUC) of ROC in the verification set was 0.808, which indicated that the model has a good degree of differentiation. Then the correction curve results showed that the prediction probability of the model in the training set and verification set was like that of the actual DF occurrence, which indicated that the prediction model was accurate. In addition, the DCA curve indicated that when the risk threshold was between 10% and 85%. The clinical net income of the patients was higher, indicating that the safe application threshold of the model was wide, the clinical practical value was high, and the data in the verification set have achieved similar results.

Previously, we successfully established a nomogram prediction model to predict the risk of amputation in patients with diabetic feet and applied it to clinical practice with remarkable results (9). But it is well known that when diabetic patients progress to diabetic foot, their prognosis is often poor, and many patients are at risk of amputation or even death. Therefore, it is particularly important to identify diabetic foot patients early in their lives. However, the models for predicting diabetic foot are rare. Based on previous work (9), we established and validated the nomogram model for predicting diabetic foot in patients with type 2 diabetes. The model showed good results. This is of great significance for the early assessment of the occurrence of DF. This model provides a very effective means for the risk factor analysis and early diagnosis of diabetic foot.

As far as we know, this is one of only a few limited nomogram models with high accuracy to visually predict the risk of DF in patients with T2DM based on clinical indicators. However, there are still several limitations to our current study. First, the sample size is limited; second, insufficient diabetic foot risk factors were included in the model; although this nomogram has achieved satisfactory accuracy, there is still a lot of room for further prospective multi-center validation to confirm and improve the reliability of this nomogram and its clinical application; third, this study was designed retrospectively; due to limited data availability, not all clinical indexes were included in the study, and further improvement is needed to explore a more comprehensive and accurate reinforcement model. In conclusion, our study further confirmed that age, smoking history, HbA1c, WBC, and LDL-C were independent risk factors affecting the occurrence of DF. Based on the above indicators, we constructed a nomogram model to predict the risk of DF in patients with T2DM. This model can effectively quantitatively evaluate the risk of DF in patients with T2DM, provides a simple and intuitive tool for clinical workers to identify people at high risk of DF, and has guiding significance for early diagnosis and individual prevention of DF patients.

## Data availability statement

The original contributions presented in the study are included in the article/supplementary material. Further inquiries can be directed to the corresponding author.

## Author contributions

BP and RM contributed equally to the manuscript.
